# Avian Influenza Seroprevalence and Biosecurity Risk Factors in Maryland Backyard Poultry: A Cross-Sectional Study

**DOI:** 10.1371/journal.pone.0056851

**Published:** 2013-02-20

**Authors:** Jennifer M. Madsen, Nickolas G. Zimmermann, Jennifer Timmons, Nathaniel L. Tablante

**Affiliations:** 1 Virginia-Maryland Regional College of Veterinary Medicine, University of Maryland, College Park, Maryland, United States of America; 2 Department of Animal and Avian Sciences, University of Maryland, College Park, Maryland, United States of America; 3 Lower Eastern Shore Research and Education Center, University of Maryland Extension, Salisbury, Maryland, United States of America,; University of Hong Kong, China

## Abstract

Major implications on a country's economy, food source, and public health. With recent concern over the highly pathogenic avian influenza outbreaks around the world, government agencies are carefully monitoring and inspecting live bird markets, commercial flocks, and migratory bird populations. However, there remains limited surveillance of non-commercial poultry. Therefore, a cross-sectional study was conducted in backyard poultry flocks using a convenience sampling method across three regions of Maryland from July 2011 to August 2011. The objective of this study was to develop a better understanding of the ecology and epidemiology of avian influenza by investigating the prevalence and seroprevalence in this potentially vulnerable population and by evaluating biosecurity risk factors associated with positive findings. Serum, tracheal, and cloacal swabs were randomly collected from 262 birds among 39 registered premises. Analysis indicated bird and flock seroprevalence as 4.2% (11/262) and 23.1% (9/39), respectively. Based on RT-qPCR analysis, none of the samples were found to be positive for AI RNA and evidence of AI hemagglutinin subtypes H5, H7, or H9 were not detected. Although no statistically significant biosecurity associations were identified (p≤0.05), AI seroprevalence was positively associated with exposure to waterfowl, pest control, and location. AI seropositive flocks exposed to waterfowl were 3.14 times as likely to be AI seropositive than those not exposed (p = 0.15). AI seropositive flocks that did not use pest control were 2.5 times as likely to be AI seropositive compared to those that did and AI seropositive flocks located in the Northern region of Maryland were 2.8 times as likely to be AI seropositive than those that were located elsewhere.

## Introduction

Avian Influenza (AI) is a type A Influenza virus and zoonotic pathogen of significant economic and public health concern. Of particular interest is the highly pathogenic avian influenza (HPAI) H5N1 subtype. Emerging in 1997, it has been responsible for the deaths of millions of birds globally and continues to persist at endemic levels in some countries [1]. The HPAI H5N1 subtype is also capable of crossing the species barriers into human populations [2]. To date, HPAI H5N1 has not been detected in the U.S., though several other HPAI and low pathogenic avian influenza (LPAI) subtypes have surfaced over the years in bird populations which have cost millions of dollars in response and recovery efforts[3], [4]. In the spring of 2004, the Delmarva Peninsula, regions of Delaware, Maryland, and Virginia, experienced an LPAI H7N2 outbreak that resulted in the culling of 378,000 birds [5], [6].

This location is of interest when it comes to AI surveillance for several reasons. Delmarva and the Chesapeake Bay coincide with the final significant merging zone of the Atlantic Migratory Flyway serving waterfowl, the natural reservoirs for influenza A viruses, from the far reaches of the Arctic Ocean, Northwest Territories of Canada, and Greenland [7]. In 1998, a survey of free flying resident ducks on the Eastern Shore of Maryland revealed that almost 14% of the sampled population was positive for AI, representing nine different subtype combinations [8]. Another study reported that shorebirds migrating through the Delaware Bay had the highest frequency of AI viruses compared to similar populations along the Atlantic flyway [9]. Delmarva is also within close proximity to the live bird markets of the Northeast, which have been susceptible to AI outbreaks in the past [10].

Disease surveillance and prevention are critical as the U.S. is the world's leading producer of poultry meat and the second largest poultry meat exporter and egg producer, valuing the industry at over $35.6 billion a year in 2010 [11]. Delmarva has a dense commercial poultry industry with over 1,500 broiler operations, placing Maryland at eighth in the nation's top broiler producing states in 2011 [12]. Ownership of backyard poultry is also becoming a fast growing trend for many Americans, which make up a diverse community with varying education and management practices. These factors support the need for ongoing surveillance research and biosecurity education to minimize the costs associated with quarantines, depopulation, loss of production time, and international trade restrictions.

At present, only a few studies have evaluated the prevalence of AI in backyard flocks. Government agencies are carefully monitoring and inspecting live bird markets, commercial flocks, and migratory bird populations. However, there remains little surveillance of private poultry flocks which are not confined to the same strict biosecurity practices as their commercial counterparts. Therefore, a cross-sectional study was conducted in non-commercial backyard poultry flocks using a convenience sampling method across three regions of Maryland from July 2011 to August 2011. The objective of this study was to investigate the prevalence and seroprevalence of avian influenza in this potentially vulnerable population and to evaluate biosecurity risk factors associated with positive findings.

## Materials and Methods

### Ethics Statement

This study was approved in accordance with the University of Maryland's Institutional Review Board (IRB #11-0335), Federal Policy for the Protection of Human Subjects (45 CFR 46), and Institutional Animal Care and Use Committee (IACUC # R-11-27). Written informed consent was obtained from all participants prior to survey and sample collection.

### Study Design and Population

This study used a cross-sectional survey design and convenience sampling method to determine biosecurity risk factors and disease prevalence among Maryland non-commercial poultry flocks. Surveillance included active observational, active serologic, and active antigen methods. Counties were chosen based on the proportion of registered backyard flock owners and location of commercial industries and auction markets. In May 2011, the Maryland Department of Agriculture (MDA) confidentially mailed 1,000 informational letters and return postcards to poultry owners enrolled in the Maryland Poultry Registration Program. Participants were eligible for the study if they lived in Maryland, owned domesticated fowl, and maintained a flock size fewer than 1,000 birds.

### Study Sites

Study sites were designated by counties within three regions of Maryland: Northern (Frederick & Carroll), Southern (St. Mary's & Charles), and Eastern Shore (Caroline, Dorchester, Talbot, Wicomico, & Worcester) (Table 1).

**Table 1:** Outline of dates, locations, and species per sampled backyard flock.

Date of Sample Collection	Flock ID	Region^a^/County	Sampled Species					Total Birds Sampled
			Chicken	Turkey	Duck	Guinea Fowl	Pheasant	
**7/15/2011**	1	(N) Frederick	6					6
	2	(N) Frederick	5					5
	3	(N) Frederick	7					7
	4	(N) Frederick	6					6
	5	(N) Frederick	12					12
**7/19/2011**	6	(N) Frederick	21					21
	7	(N) Frederick	3					3
	8	(N) Frederick	8					8
	9	(N) Frederick	2	2	2			6
	10	(N) Frederick	6					6
**7/21/2011**	11	(S) St. Mary's	2	2		2		6
	12	(S) St. Mary's	3					3
	13	(S) St. Mary's	6					6
	14	(S) St. Mary's	4	2				6
	15	(S) St. Mary's	4		2			6
	16	(S) St. Mary's	6					6
**7/26/2011**	17	(E) Wicomico	3					3
	18	(E) Wicomico	10					10
	19	(E) Wicomico	6					6
	20	(E) Wicomico	3					3
	21	(E) Wicomico	6					6
**7/28/2011**	22	(N) Frederick	6					6
	23	(N) Frederick	4		1			5
	24	(N) Frederick	8					8
	25	(N) Frederick	6					6
	26	(N) Frederick	6					6
**8/1/2011**	27	(S) Charles	8					8
	28	(S) Charles	4		2			6
	29	(S) Charles	4		2		2	8
	30	(S) Charles	2		4			6
**8/3/2011**	31	(E) Dorchester	4					4
	32	(E) Talbot	4	4				8
	33	(E) Caroline	6					6
	34	(E) Talbot	4					4
**8/25/2011**	35	(N) Frederick	10	6	2			18
	36	(N) Carroll	6					6
	37	(N) Carroll	6					6
	38	(N) Carroll	4					4
	39	(N) Frederick	6					6
**Total**			**227**	**16**	**15**	**2**	**2**	**262**

a *Region abbreviations (N = North, S = South, E = East).*

### Biosecurity Questionnaire

Upon state and academic review, a four page questionnaire and information sheet was mailed to backyard flock owners. Participants were asked to self-report information on the number and species of poultry reared, presence of other animals, animal husbandry, opportunities for interaction between wild birds and poultry, flock biosecurity measures, and health status of poultry. Questionnaire is available upon request.

### Sample Collection

Blood (1–3 ml) was collected from the brachial vein of each bird and placed in a serum separator vacutainer. Tracheal and cloacal swabs were also collected, and stored in vials containing 2.5 ml of protein based brain-heart infusion (BHI) transport media. All tubes were labeled with date, species, sample type, and location. Once samples were collected, they were stored at 4°C (24–48 hours) until processed.

### Serologuc Assays

#### cELISA

Serum was separated from the clot by centrifugation at 1,300× g for 10 minutes in a swinging bucket centrifuge and stored at −20°C. Evaluation for antibodies to influenza A viruses in sera was carried out using Synbiotics USDA-licensed screening kit, *Flu* DETECT® BE. The *Flu* DETECT® BE kit is designed to detect antibodies against a recombinant nucleoprotein. Plates were read using the ELX800 microplate reader (BIO-TEK instruments, INC., Winooski, VT) and ProFILE3 software (Synbiotics Corp., Kansas City, MO). Positive serum was determined based on the serum sample to negative control ratio (SN<0.6) designated by the Synbiotics kit. SN<0.6 is equivalent to 40% inhibition.

### Viruses

Influenza virus strains A/Mallard/PA/10218/84 (H5N2), A/Mallard/Alberta/24/01 (H7N3), and A/Quail/Arkansas/20209-1/93 (H9N2) were generously provided by Dr. Daniel Perez from the University of Maryland (College Park, MD). Viruses were propagated in nine day-old embryonated chicken eggs for 48 hours as previously described [13].

### Hemagglutination (HA) and Hemagglutination Inhibition (HI) Assays

HA titers were determined using 50 ul of 0.5% chicken red blood cells in PBS to 50 ul of a two-fold serial dilution of virus and PBS. Microtiter plates were incubated for 30 minutes at room temperature. HA titers were subsequently calculated as the reciprocal value of the highest dilution that caused complete hemagglutination. HI titrations were calculated by performing a serial two-fold dilution of 25 ul of Receptor Destroying Enzyme (RDE) treated sample and control serum with 25 ul of PBS. Twenty five ul of virus dilution containing 4 HA units/25 ul was then added to each well. Wells were incubated at room temperature for 30 minutes and 50 ul of 0.5% chicken red blood cell suspension was added. After 30 minutes HI titers were calculated as the reciprocal of the serum dilution that inhibited hemagglutination. A titer of 1∶128 was used to define the reactivity of samples. This was the titer of the last well in a serial dilution of the positive control column that completely inhibited hemagglutination [14].

### Antigen Assays

#### RNA Purification

Swabs were removed from the BHI transport media and samples vortexed for 5 seconds followed by centrifugation for 5 minutes at 5,000× g. Supernatant was processed following the organic method protocol [15]. RNA samples were stored at −80°C while awaiting RT-qPCR analysis.

### Reverse Transcription Quantitative PCR (RT-qPCR)

RT-qPCR was conducted on the Bio-Rad (Hercules, CA) CFX96 Real-Time thermal cycler and analyzed with CFX Manager Software using the one-step QuantiTect SYBR® green RT-PCR kit (Qiagen, Valencia, CA). For gallinaceous poultry (chickens, turkey, quail, pheasant) tracheal RNA swab samples were used for AIV RT-qPCR analysis as these viruses primarily replicate in the respiratory tract. For waterfowl, cloacal RNA swab samples were used as AI virus primarily replicates in the intestinal tract of these birds [16]. Duplicate samples were prepared using a specific matrix gene primer M+25 (5′-AGA TGA GTC TTC TAA CCG AGG TCG-3′) and M-124 (5′-TGC AAA AAC ATC TTC AAG TCT CTG-3′) [15]. Chicken GAPDH specific primers were also included on each 96 well plate as an internal control GAPDH+223 (5′- GGC ACT GTC AAG GCT GAG AA-3′) and GAPDH-321 (5′- TGC ATC TGC CCA TTT GAT GT-3′) [17]. Reaction mixtures included 10 ul of 1× QuantiTect SYBR Green RT-PCR Master Mix, 0.5 ul each of forward and reverse primers of 10 uM concentration (IDT), 0.2 ul of QuantiTect RT mix, 2.3 ul of nuclease free water, 0.5 ul of RNase inhibitor (13Units/ul) (RNasin, Promega), and 6 ul of RNA extract, for a total reaction volume of 20 ul. Samples were incubated at 50°C for 30 minutes, 95°C for 15 minutes followed by 40 cycles of 94°C at 15 seconds, 60°C at 30 seconds, and 72°C at 30 seconds. A melt curve analysis was conducted with each run. Positive, no template, and no enzyme controls were included on each plate as well.

### Statistical Analysis

After descriptive data analysis (mean, median, and range), univariate and multivariate statistical analyses were carried out. The association of the independent variables elucidated from the questionnaire, such as biosecurity practices and the dependent variables (bird or flock disease positive) were analyzed using Fisher's exact test, (right sided) for the categorical variables due to small counts (Table 2). Disease status and independent variables of each flock were coded into a binary outcome (Disease = 1, No disease = 0) and (Exposed = 1, Not exposed = 0). Strengths of associations were also calculated and reported as relative risks. Relative risk is the ratio of the probability of disease occurring in the exposed group versus the non-exposed group. Continuous variables were analyzed by simple logistic regression (Table 3). A p-value<0.25 was set as the inclusion threshold for categorical and continuous variables into multivariate analysis. Multiple logistic regression containing all continuous and categorical variables with a p-value<0.25 was executed for selection into a final stepwise backward elimination regression model. Variables with a p-value<0.05 were considered statistically significant for association with the outcome. Data were analyzed using Statistical Analysis System (SAS) software for Windows v9.2 (SAS Institute, Cary, NC) and Statistix9 for Windows (Analytical Software, Tallahassee, FL).

**Table 2:** Categorical variables examined for association with AI seropositive flocks.

Biosecurity risk factor	Description
Housing (HOUSING)	Free range vs. coop
Species Separate (SPECSEP)	Together vs. separate
Owner exp wild waterfowl (OWNWFOWL)	Exposed vs. not exposed
Owner exp wild birds (OWNWDBRD)	Exposed vs. not exposed
Owner exp neighbor birds (OWNNEBRD)	Exposed vs. not exposed
Owner exp rodents (OWNRODNT)	Exposed vs. not exposed
Owner exp wild carnivore (OWNCARN)	Exposed vs. not exposed
Owner exp livestock (OWNLVSTK)	Exposed vs. not exposed
Bird exp wild waterfowl (BRDWFOWL)	Exposed vs. not exposed
Bird exp wild birds (BRDWDBRD)	Exposed vs. not exposed
Bird exp pets (BRDPETS)	Exposed vs. not exposed
Bird exp rodents (BRDRODNT)	Exposed vs. not exposed
Bird exp wild carnivore (BRDCARN)	Exposed vs. not exposed
Bird exp livestock (BRDLVSTK)	Exposed vs. not exposed
Allow visitors (ALLVIS)	Allow visitors vs. no visitors
Isolate new birds (ISONWBRD)	No isolation vs. isolation
Disease mortality (DIESICK)	Deaths vs. no deaths
Diarrhea (DIARRHEA)	Sick vs. not sick
Respiratory disease (RESPDIS)	Sick vs. not sick
Neurologic disease (NEURODIS)	Sick vs. not sick
Weight loss (WGTLOSS)	Sick vs. not sick
Footbath/footwear (FOOTBATH)	No footbath vs. footbath
Clean and disinfect (CLEAN)	Don't clean vs. do clean
Pest control (PESTCON)	No pest control vs. pest control
Region (REGION)	North, South, or East vs. other regions

**Table 3:** Continuous variables examined for association with AI seropositive flocks.

Biosecurity risk factor	Description
Commercial farms (COMMFARM)	Number of farms within 1/4 mile
Backyard flocks (BACKFLCK)	Number of backyard flocks within 1/4 mile
Years of ownership (YEAROWN)	Number of years kept poultry
Flock size (FLCKSZE)	Number of birds in flock
Visit commercial (VISCOMM)	Number of times visit commercial farm (1 yr)
Visit backyard flocks (VISBKYD)	Number of times visit backyard flock (1 yr)

## Results

**Figure 1:**
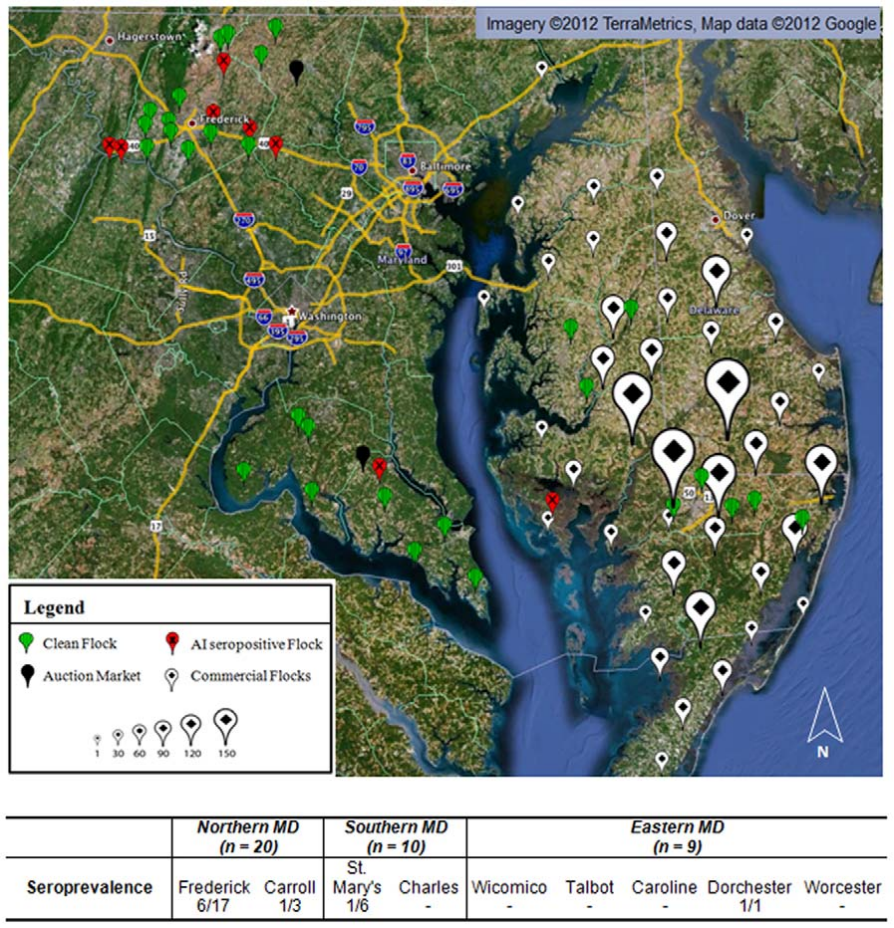
Sample site locations with AI seropositive backyard flocks. Poultry were grouped by size based on number of commercial houses within a 15 km radius.

The overall survey response rate was 4.1% (41/1000). Two backyard flock owners of the 41 could not be reached for testing arrangements. From July 15–August 25, 2011, 262 birds from 39 backyard flocks were sampled. The sampled poultry population consisted of various ages and species including 227 chickens (*Gallus domesticus*), 16 turkeys (*Meleagris gallopavo*), 15 ducks (*Anas platyrhynochos, Cairina moschata*), 2 guinea fowl (*Numida meleagris*), and 2 pheasants (*Phasianus colchicus*). Seroprevalence of AI in backyard birds was 4.2% (11/262), while the overall flock seroprevalence was 23.1% (9/39) (Table 4). HI tests did not detect H5, H7, or H9 subtype-specific antibodies among the ELISA-positive sera. Chickens were the only species that were seropositive on nine premises among four counties. One bird from each premises was seropositive except one Frederick and one St. Mary's flock which had two seropositive birds each (Fig. 1). Based on RT-qPCR analysis, none of the samples were found to be positive for AI RNA. All seropositive flocks were reported and subsequently tested by the MDA, all of which were determined to be negative for current infection.

**Table 4:** Results of antigen and serological screenings of 262 birds from 39 backyard flocks.

*Titer Distribution*				*Seropositive birds/total birds*	*Seropositive flocks/total flocks*	*Positive flocks/total flocks*
*<1,000*	*1,000–1,999*	*2,000–3,999*	*≥4,000*	11/262 (4.2%)	9/39 (23.1%)	0
7	2	2	0			

In this cross-sectional study we also evaluated transmission pathways and biosecurity risk factors that may be associated with seropositives. Of the 39 flocks sampled, 36 completed the survey and were analyzed for statistically significant associations. No significant associations (p≤0.05) were identified; however, some risk factors showed a positive association after relative risk calculations. 67% (2/3) of seropositive flocks were exposed to waterfowl compared to 21% (7/33) that were not exposed. Seropositive flocks exposed to waterfowl were therefore 3.14 times as likely to be AI seropositive than those not exposed to waterfowl (95% confidence interval [C.I.] = 1.1–8.9; p = 0.15). 33% (7/21) of seropositive flocks did not use pest control compared to 13% (2/15) that did. Seropositive flocks that did not use pest control were 2.5 times as likely to be AI seropositive than those that did (C.I. = 0.6–10.4; p = 0.17). 35% (7/20) of seropositive flocks were from Northern Maryland while 13% (2/16) were from other regions. Seropositive birds from Northern Maryland were 2.8 times as likely to be AI seropositive than those from Southern or Eastern Maryland (C.I. = 0.7–11.7; p = 0.12). Five out of 11 flocks (46%) that were AI seropositive had also experienced diarrhea in the past six months compared to 16% (4/25) of AI seropositive flocks that did not exhibit diarrhea. Seropositive flocks that experienced diarrhea within the past six months were 2.8 times more likely to be AI seropositive than those that did not experience diarrhea (C.I. = 0.9–8.6; p = 0.08). Results from statistical analysis may be found in Tables 5, 6, and 7.

**Table 5:** Univariate analysis of categorical biosecurity variables (P≤0.25).

Variable	Description	Prevalence Ratio	95% Confidence Interval	P-value
Diarrhea	Reported within past 6 mo.	2.84	0.939–8.596	0.075
Location	North vs. other regions	2.80	0.672–11.670	0.122
Pest control	Implemented pest control	2.50	0.601–10.394	0.165
Waterfowl	Exposed to wild waterfowl	3.14	1.116–8.853	0.148

**Table 6:** Multivariate logistic regression (P≤0.25).

Variable	Description	Coefficient	P-value
Time owned	How many years kept poultry	0.613	0.133
Visit comm.	How often visit commercial sites	2.701	0.104
Diarrhea	Reported within past 6 mo.	−1.314	0.380
Location	North vs. other regions	2.500	0.204
Pest control	Implemented pest control	−0.107	0.942
Waterfowl	Exposed to wild waterfowl	18.377	0.736

**Table 7:** Backward selection stepwise logistic regression model to examine association between biosecurity risk factors and AI seroprevalence (P≤0.05).

Variable	Description	Coefficient	P-value
Time owned	How many years kept poultry	0.154	0.127
Visit comm.	How often visit commercial sites	0.713	0.080
Location	North vs. other regions	2.379	0.102

## Discussion

This study suggests that backyard flocks are no exception to avian influenza exposure and that Maryland flocks may have been exposed to AI from wild birds or pests. Pests are defined as both mammals and invertebrates. AI vaccination was ruled out based on survey data, as all owners denied vaccinating flocks once on the premises. AI vaccination practices are also rare in the U.S. and require USDA licensure and approval from both state and federal governments prior to field deployment [18]. To date, only a handful of studies based in industrialized countries have evaluated the seroprevalence of avian influenza in unvaccinated backyard flocks. While one study in New Zealand found a flock seroprevalence of 20.8% (5/24), comparable to 23.1% (9/39) in this study, a Minnesota team only detected one flock out of 150 (0.66%) for AI antibodies [19], [20]. In Switzerland, researchers reported a higher seroprevalence of AI at 37.5% (15/40) in fancy breeding flocks [21]. However, many variables contribute to sample prevalence rates such as testing method, time of year, climate differences, migratory trends, species and age of waterfowl, and backyard flock exposure and management practices.

Earlier studies focusing on the Delaware Bay and Maryland's Eastern shore have shown the prevalence of AI reservoir species ranging from May to November. The Delaware Bay has been identified as a “hotspot” for AIV prevalence, from May to June, in shore birds, particularly the ruddy turnstone, however, the surveying time period excludes this population. Migratory waterfowl also travel up the Atlantic Flyway and arrive late July through October with peak AIV prevalence detected in August [22], [23]. A study on the Eastern Shore of Maryland sampled cloacal swabs from resident ducks for 3 weeks between May 28 and Sept 2, 1998. Results suggested that influenza A viruses were introduced or increased in prevalence in resident waterfowl between July 15 and Aug 27 as AIV positives were detected from August 27 to September 2 at a prevalence of 13.9% [8].

While no AI RNA was detected in backyard poultry flocks, serological analysis indicated that almost a quarter of flocks had been previously exposed. Detection of antibodies against AI also allowed for screening of poultry that were infected prior to the sampling period. Detectable levels of antibodies against AI appear one to two weeks after infection and can last for several months [24]. Sera positive for antibodies were also screened for hemagglutinin (HA) subtypes H5, H7, and H9 which are thought to have the greatest pandemic potential by the World Health Organization as they, although rare, are transmissible from birds to humans [25]. However, these HA subtype specific antibodies were not found in this study which is consistent with other publication findings. Previous influenza surveillance studies conducted in Maryland waterfowl have reported the presence of HA subtypes H2, H3, H6, H9, H11, and H12, whereas the majority of North American subtypes consist of H3, H4, and H6 [8], [9], [26].

It is believed that all of the AI seropositive chickens identified in this study were exposed to LPAI viruses as the birds survived the infection and owners did not report any significant mortalities in their flocks as a result of disease. The majority of circulating strains are low pathogenic viruses which may produce subtle or no signs of clinical infection to mild respiratory distress. Other signs may include diarrhea, decrease in egg production, and inactivity. However, these signs are not specific to AI infection and are often present in other poultry diseases [3], [27]. Almost half of owners (46%) with an AI positive test observed diarrhea in their flock within the past six months. One third of AI seropositive flock owners reported a decrease in egg production or soft/misshapen eggs in the previous six months and only one AI seropositive flock exhibited coughing, sneezing, nasal secretions, or swollen sinuses. Another indication that flocks may have been exposed to LPAI viruses was the negative HI assay result for H5 and H7 influenza subtypes, which are the exclusive subtypes associated with naturally occurring virulent isolates [28]. The lack of a secure housing environment and location near water sources, which serve as a congregation point for wild birds, waterfowl, and pests, increases the likelihood of disease transmission. These potential risks associated with disease reservoirs and vectors are similar with findings from other studies. For example, wild birds most frequently reported visiting poultry houses were sparrows and European starlings, both of which are susceptible to experimental highly pathogenic H5N1 infection and excrete high viral titers [29]. Another study conducted in an artificial barnyard setting found that mallards recently infected with H5N2 and H7N3 could transmit influenza A virus to chickens, blackbirds, rats, and pigeons demonstrating the potential for disease to spread by wild birds and pests [30]. All owners of AI seroconverted flocks, as well as most AI seronegative flocks, also allowed visitors onto their poultry premises. A higher volume of traffic on the premises potentially increases the risk of introducing disease via fomites as visitors' vehicles, boots, and clothing may carry pathogens. Several outbreak investigations have linked fomites in connection with disease spread, such as the 1983 HPAI H5N2 outbreak in Pennsylvania and Virginia commercial poultry which was associated with human and equipment traffic from New York live bird markets [31].

To the authors' knowledge, this is the first study to report associations between biosecurity management practices and disease prevalence/seroprevalence of AI among backyard flocks located within close proximity to the Delmarva commercial poultry region. However, this study was subject to some limitations. The overall response rate of this study (4.1%) was relatively poor, but believed to stem from the concern over the mandatory reporting of flock positives to the State Veterinarian and potential repercussions, such as “Hold Orders” that restrict the movement of birds onto or off the premises, as well as the stigma attached to having an infectious disease. A larger sample size may have also increased the ability of this study to detect significant associations between biosecurity risk factors and disease prevalence. While association could be hypothesized based on proportional analysis, wide confidence intervals indicate that these estimates have low precision from an inadequate sample size and therefore associated risk results should be interpreted cautiously in this preliminary study. Although methods of convenience sampling are often assumed to be representative of a population, sampling biases (most notably selection bias) do occur, making it difficult to develop statistically valid estimates of disease prevalence, regardless of how many birds are sampled. Another constraint was the lack of detail collected in the wild bird-domestic poultry interface such as type of wild bird/waterfowl species identified on the property as well as the means of exposure (i.e. nose to nose, adjacent habitat, droppings only) which may have provided greater insight to the exposure risk and should be included in future studies. Widening the sample collection time frame from May to October could have improved the chances of obtaining a more representative data set in relation to the transmission of AI from wild birds to poultry. This study was also limited to a population of backyard flock owners that had registered with the MDA. It is believed that AI prevalence estimates reported in this study are lower than the true population as most owners with clinically ill birds would be reluctant to participate. Due to the low response rate and potential biases, this study cannot be generalized to other backyard flock populations.

Surveillance is a dynamic process that requires continuous observation, collection, and analysis of data in order to identify the presence of a disease and contain its spread. While migratory waterfowl have been the main target of disease investigations, domesticated poultry warrant consideration as well. This surveillance study aimed to capture the prevalence and seroprevalence of AI during an outbreak-free period and to illustrate baseline levels of exposure in this growing population. As a result, data from this project has provided a better understanding of AI ecology and transmission relationships within backyard flocks. As demonstrated in this study, education is essential for backyard flock owners especially with non-commercial poultry ownership's recent increase in popularity. Several flock owners did not practice biosecurity methods, many of which are simple, practical, and affordable. Therefore, it is recommended that proactive biosecurity education highlight prevention measures such as protecting poultry from wild birds and waterfowl particularly during the spring and summer months when migration season is at its peak and implementing a pest control plan. Targeted education and surveillance strategies will help protect the health of U.S. poultry flocks, minimize economic effects of the disease, and greatly reduce the health risks to the U.S. public.
